# APOE-mediated suppression of the lncRNA *MEG3* protects human cardiovascular cells from chronic inflammation

**DOI:** 10.1093/procel/pwad017

**Published:** 2023-04-03

**Authors:** Hongkai Zhao, Kuan Yang, Yiyuan Zhang, Hongyu Li, Qianzhao Ji, Zeming Wu, Shuai Ma, Si Wang, Moshi Song, Guang-Hui Liu, Qiang Liu, Weiqi Zhang, Jing Qu

**Affiliations:** Division of Life Sciences and Medicine, School of Life Sciences, University of Science and Technology of China, Hefei 230001, China; State Key Laboratory of Stem Cell and Reproductive Biology, Institute of Zoology, Chinese Academy of Sciences, Beijing 100101, China; University of Chinese Academy of Sciences, Beijing 100049, China; CAS Key Laboratory of Genomic and Precision Medicine, Beijing Institute of Genomics, Chinese Academy of Sciences and China National Center for Bioinformation, Beijing 100101, China; Sino-Danish College, University of Chinese Academy of Sciences, Beijing 101408, China; State Key Laboratory of Stem Cell and Reproductive Biology, Institute of Zoology, Chinese Academy of Sciences, Beijing 100101, China; State Key Laboratory of Membrane Biology, Institute of Zoology, Chinese Academy of Sciences, Beijing 100101, China; Beijing Institute for Stem Cell and Regenerative Medicine, Beijing 100101, China; University of Chinese Academy of Sciences, Beijing 100049, China; State Key Laboratory of Membrane Biology, Institute of Zoology, Chinese Academy of Sciences, Beijing 100101, China; University of Chinese Academy of Sciences, Beijing 100049, China; State Key Laboratory of Membrane Biology, Institute of Zoology, Chinese Academy of Sciences, Beijing 100101, China; State Key Laboratory of Stem Cell and Reproductive Biology, Institute of Zoology, Chinese Academy of Sciences, Beijing 100101, China; Institute for Stem Cell and Regeneration, Chinese Academy of Sciences, Beijing 100101, China; State Key Laboratory of Membrane Biology, Institute of Zoology, Chinese Academy of Sciences, Beijing 100101, China; Beijing Institute for Stem Cell and Regenerative Medicine, Beijing 100101, China; University of Chinese Academy of Sciences, Beijing 100049, China; Institute for Stem Cell and Regeneration, Chinese Academy of Sciences, Beijing 100101, China; State Key Laboratory of Membrane Biology, Institute of Zoology, Chinese Academy of Sciences, Beijing 100101, China; Beijing Institute for Stem Cell and Regenerative Medicine, Beijing 100101, China; Advanced Innovation Center for Human Brain Protection, National Clinical Research Center for Geriatric Disorders, Xuanwu Hospital Capital Medical University, Beijing 100053, China; Aging Translational Medicine Center, International Center for Aging and Cancer, Beijing Municipal Geriatric Medical Research Center, Xuanwu Hospital, Capital Medical University, Beijing 100053, China; University of Chinese Academy of Sciences, Beijing 100049, China; Institute for Stem Cell and Regeneration, Chinese Academy of Sciences, Beijing 100101, China; State Key Laboratory of Membrane Biology, Institute of Zoology, Chinese Academy of Sciences, Beijing 100101, China; Beijing Institute for Stem Cell and Regenerative Medicine, Beijing 100101, China; University of Chinese Academy of Sciences, Beijing 100049, China; Institute for Stem Cell and Regeneration, Chinese Academy of Sciences, Beijing 100101, China; State Key Laboratory of Membrane Biology, Institute of Zoology, Chinese Academy of Sciences, Beijing 100101, China; Beijing Institute for Stem Cell and Regenerative Medicine, Beijing 100101, China; Advanced Innovation Center for Human Brain Protection, National Clinical Research Center for Geriatric Disorders, Xuanwu Hospital Capital Medical University, Beijing 100053, China; Aging Translational Medicine Center, International Center for Aging and Cancer, Beijing Municipal Geriatric Medical Research Center, Xuanwu Hospital, Capital Medical University, Beijing 100053, China; Aging Biomarker Consortium, Beijing 100101, China; Division of Life Sciences and Medicine, School of Life Sciences, University of Science and Technology of China, Hefei 230001, China; Division of Life Sciences and Medicine, Institute on Aging and Brain Disorders, The First Affiliated Hospital of USTC, Hefei National Laboratory for Physical Sciences at the Microscale, University of Science and Technology of China, Hefei 230026, China; Neurodegenerative Disease Research Center, University of Science and Technology of China, Hefei 230026, China; CAS Key Laboratory of Brain Function and Disease, University of Science and Technology of China, Hefei 230026, China; Center for Excellence in Animal Evolution and Genetics, Chinese Academy of Sciences, Kunming 650201, China; University of Chinese Academy of Sciences, Beijing 100049, China; CAS Key Laboratory of Genomic and Precision Medicine, Beijing Institute of Genomics, Chinese Academy of Sciences and China National Center for Bioinformation, Beijing 100101, China; Sino-Danish College, University of Chinese Academy of Sciences, Beijing 101408, China; Aging Biomarker Consortium, Beijing 100101, China; Division of Life Sciences and Medicine, School of Life Sciences, University of Science and Technology of China, Hefei 230001, China; State Key Laboratory of Stem Cell and Reproductive Biology, Institute of Zoology, Chinese Academy of Sciences, Beijing 100101, China; University of Chinese Academy of Sciences, Beijing 100049, China; Institute for Stem Cell and Regeneration, Chinese Academy of Sciences, Beijing 100101, China; Beijing Institute for Stem Cell and Regenerative Medicine, Beijing 100101, China; Aging Biomarker Consortium, Beijing 100101, China

**Dear Editor,**

Cardiovascular diseases (CVDs) are the leading cause of death world-wide. Thus, diagnosing and treating CVD remains at the forefront for clinicians while identifying targetable disease mechanisms in preclinical models are focus areas for researchers and drug developers (Cai et al., 2022a). The polymorphic protein apolipoprotein E (APOE), central to lipid transport and metabolism, is well-recognized for the role of its isoforms as important predictors for human cardiovascular disorders and neurodegenerative diseases (Tudorache et al., 2017). Plasma APOE is generated primarily from liver hepatocytes, accounting for around 75% of the APOE production from the whole body (Getz and Reardon, 2009), and plays important functional roles in monocytes/macrophages, adipocytes, and the central nervous system (Kockx et al., 2018). However, despite the fact that APOE is widely expressed in different mammalian cells, studies on the functional roles of APOE mostly focus on its extracellular secreted form, and the specific effects of APOE, particularly intracellular form in cell types closely related to human cardiovascular diseases are therefore still poorly understood.

To explore the role of APOE in human heart and blood vessel cells, we derived human vascular endothelial cells (hVECs), human vascular smooth muscle cells (hVSMCs) and human cardiomyocytes (hCMs) from APOE-deficient (*APOE*^−/−^) human embryonic stem cells (hESCs) (Fig. 1A). As controls, we also generated healthy cardiovascular cells from wild-type (WT, *APOE*^+/+^) hESCs (Fig. 1A). We first confirmed that APOE expression was absent in the differentiated human cell derivatives (Fig. 1B). Then, by immunofluorescence or flow cytometric analysis, we validated the identities of the differentiated cell types. Among these, we purified hESCs-derived hVECs using fluorescence-activated cell sorting (FACS) with antibodies recognizing endothelial cell-specific markers CD31 and CD144 (Fig. S1A) and validated that 99.9% of the derived hVECs were double positive for immunostaining of the endothelial cell-specific markers CD31 and von Willebrand Factor (vWF) (Fig. S1B). Similarly, 99.9% of the derived hVSMCs expressed the smooth muscle cell-specific markers CD140b, Calponin, SM22 (Fig. S1C and S1D), and 99.8% of the differentiated hCMs expressed the cardiomyocyte-specific marker cardiac troponin T (cTnT) (Fig. S1E). Across these three cell types, the differentiation efficiencies of *APOE*^+/+^ and *APOE*^−/−^ hESCs were comparable, indicating that APOE is dispensable for the differentiation of hESCs into cardiovascular cells.

**Figure 1.**
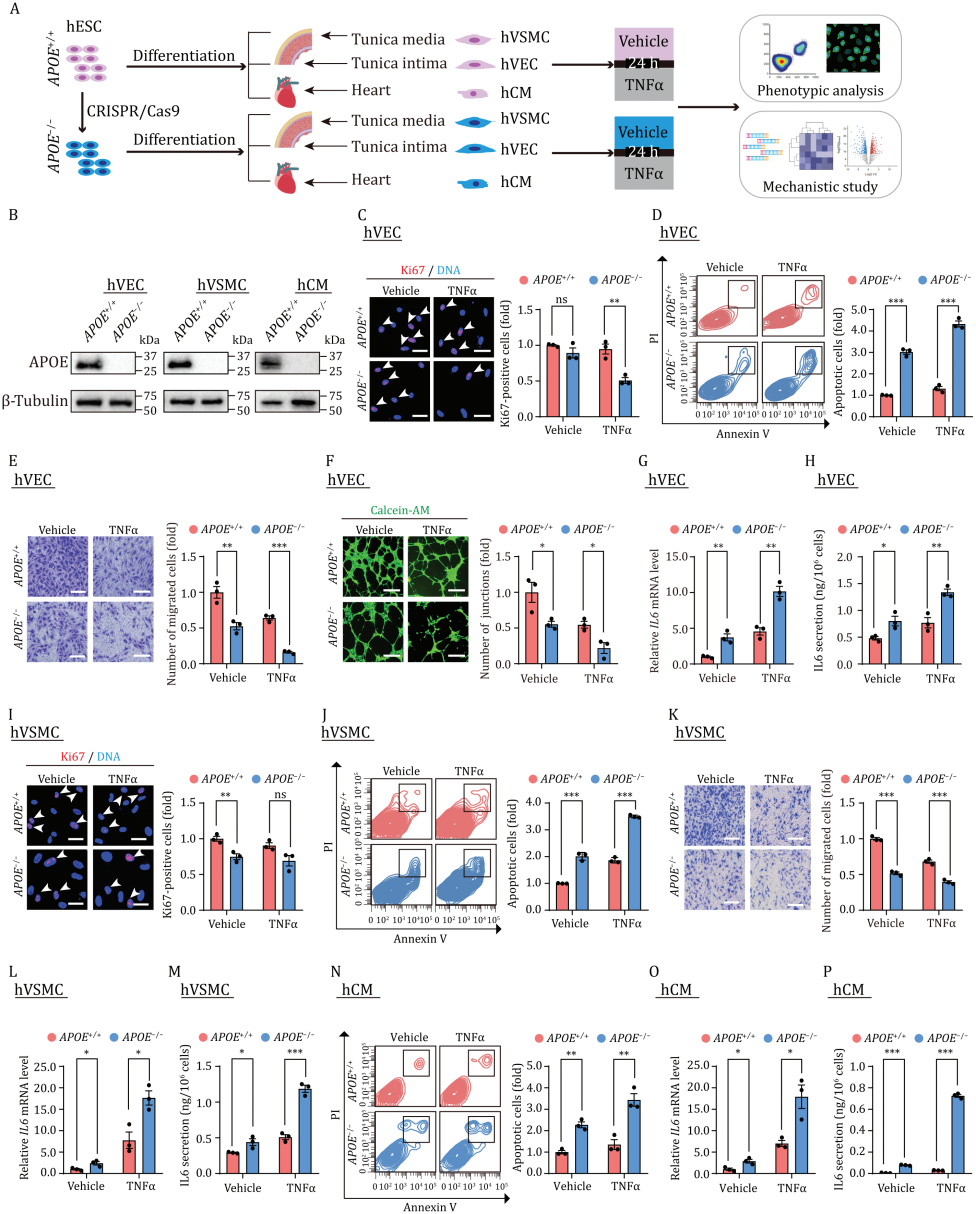
**APOE is critical for maintaining survival and function of cardiovascular cells.** (A) The schematic diagram of the workflow, phenotype analysis and mechanistic study for hVECs were performed at passage 3, and those for hVSMCs and hCMs were performed at passage 2. (B) Western blot analysis of APOE across three cell types, β-Tubulin was used as the loading control. (C) Immunofluorescence analysis of Ki67 in hVECs with or without TNFα treatment. Scale bar, 25 μm. The white arrows indicate Ki67-positive cells. Statistical results are presented as the means ± SEM. *n* = 3 independent experiments. ns, not significant, **P < 0.01. (D) Flow cytometry-based apoptosis detection of hVECs with or without TNFα treatment. Statistical results are presented as the means ± SEM. *n* = 3 biological repeats. ***P < 0.001. (E) Cell migration assay of hVECs with or without TNFα treatment. Scale bar, 100 μm. Statistical results are presented as the means ± SEM. *n* = 3 independent experiments. **P < 0.01, ***P < 0.001. (F) Tube formation assays of hVECs with or without TNFα treatment. Scale bar, 100 μm. Statistical results are presented as the means ± SEM. *n* = 3 independent experiments. *P < 0.05. (G) qRT-PCR analysis of the expression levels of *IL6* in hVECs with or without TNFα treatment. Statistical results are presented as the means ± SEM. *n* = 3 independent experiments. **P < 0.01. (H) ELISA analysis for the secreted IL6 in culture medium of hVECs with or without TNFα treatment. Statistical results are presented as the means ± SEM. *n* = 3 biological repeats. *P < 0.05, **P < 0.01. (I) Immunofluorescence analysis of Ki67 in hVSMCs with or without TNFα treatment. Scale bar, 25 μm. The white arrows indicate Ki67-positive cells. Statistical results are presented as the means ± SEM. *n* = 3 independent experiments. ns, not significant, **P < 0.01. (J) Flow cytometry-based apoptosis detection of hVSMCs with or without TNFα treatment. Statistical results are presented as the means ± SEM. *n* = 3 biological repeats. ***P < 0.001. (K) Cell migration assays of hVSMCs with or without TNFα treatment. Scale bar, 100 μm. Statistical results are presented as the means ± SEM. *n* = 3 independent experiments. ***P < 0.001. (L) qRT-PCR analysis of the expression levels of *IL6* in hVSMCs with or without TNFα treatment. Statistical results are presented as the means ± SEM. *n* = 3 independent experiments. *P < 0.05. (M) ELISA analysis for the secreted IL6 in culture medium of hVSMCs with or without TNFα treatment. Statistical results are presented as the means ± SEM. *n* = 3 biological repeats. *P < 0.05, ***P < 0.001. (N) Flow cytometry-based apoptosis detection of hCMs with or without TNFα treatment. Statistical results are presented as the means ± SEM. *n* = 3 biological repeats. **P < 0.01. (O) qRT-PCR analysis of the expression levels of *IL6* in hCMs with or without TNFα treatment. Statistical results are presented as the means ± SEM. *n* = 3 independent experiments. *P < 0.05. (P) ELISA analysis for the secreted IL6 in culture medium of hCMs with or without TNFα treatment. Statistical results are presented as the means ± SEM. *n* = 3 biological repeats. ***P < 0.001.

Unresolved chronic low-grade inflammation is considered as a key driver for cardiovascular aging and the development of aging-related diseases, and pro-inflammatory factors such as tumor necrosis factor-α (TNFα), interleukins (ILs), which activate the immune response of the cardiovascular cells and induce cellular dysfunction, coalesce to cause cardiovascular dysfunctions (Cai et al., 2022b; Zhang et al., 2022b). In particular, TNFα is among the most common pro-inflammatory cytokines observed following cardiovascular injuries (Lei et al., 2021). Here, we treated *APOE*^+/+^ and *APOE*^−/−^ human cardiovascular cells with TNFα to mimic the inflammatory milieu and performed a series of functional assays (Fig. 1A). TNFα treatment resulted in a nearly 50% decrease in the ratio of Ki67-positive cells in *APOE*^−/−^ hVECs compared to that in *APOE*^+/+^ hVECs (Fig. 1C). In contrast, under normal culture condition, the absence of APOE did not lead to obvious changes in the proliferation ability of hVECs (Fig. 1C). Concurrently, the colony-forming ability was largely abolished upon TNFα treatment, especially in *APOE*^−/−^ hVECs (Fig. S2A). These data indicated that APOE is required for hVEC proliferation upon inflammatory injury.

When we performed flow cytometric analysis, we found that APOE-deficient hVECs were more prone to apoptosis, and this vulnerability was further aggravated in the presence of TNFα treatment (Fig. 1D). In addition, the cell migration ability of *APOE*^−/−^ hVECs was markedly reduced when compared to control cells (Fig. 1E), as reflected by the number of migrating cells reduced to nearly half of the number of migrating *APOE*^+/+^ hVECs. Endothelial cell migration is essential to angiogenesis, which is commonly assessed by *in vitro* formation of capillary-like tubes from hVECs (Zhang et al., 2020; Wang et al., 2022a). As expected, tube formation in *APOE*^−/−^ hVECs was impaired and was further exacerbated along with reduced migration ability after TNFα treatment (Fig. 1F). In addition, expression of inflammatory factors such as interleukin6 (*IL6*) and monocyte chemotactic protein-1 (*MCP1*) were all increased in *APOE*^−/−^ hVECs, and in the presence of TNFα treatment, we observed synergistically promoted expression of inflammatory factors (Figs. 1G, 1H and S2B). Together, these results indicate that APOE is critical for maintaining hVEC survival and function in both physiological and inflammatory injury contexts.

In hVSMCs, we found that APOE depletion disrupted cell homeostasis, as manifested by a decreased number of Ki67-positive cells and compromised capacity to form single-cell colonies (Figs. 1H and S2C). Although TNFα treatment did not further exacerbate the proliferative impairment of *APOE*^−/−^ hVSMCs (Figs. 1I and S2C), other defects were aggravated upon TNFα treatment, including augmented cell apoptosis, and compromised cell migration (Fig. 1J and 1K). Consistent with the observed phenotypes, the levels of pro-inflammatory factors were all further increased in *APOE*^−/−^ hVSMCs in the presence or absence of TNFα treatment (Figs. 1L, 1M and S2D). Taken together, our data demonstrate that APOE is a key vasoprotective factor that safeguards human vascular cells against inflammatory injuries, and that APOE deficiency leads to disruption of homeostasis and reduced stress tolerance.

Cardiomyocytes (CMs) are post-mitotic cells lacking proliferative potential (Wang et al., 2022b; Zhang et al., 2022a). Therefore, we mainly focused on evaluating the effects of APOE deficiency on the viability of hCMs. Our data showed that APOE deletion induced hCMs apoptosis, which was even more severe than that induced by TNFα in *APOE*^+/+^ hCMs (Fig. 1N). Interestingly, IL6 secretion was markedly induced in APOE-depleted hCMs and in hCMs treated with TNFα. APOE depletion synergized with TNFα treatment to induce a nearly thousand-fold increase in IL6 secretion levels (Figs. 1O, 1P and S2E). Quantitative real-time PCR (qRT-PCR) further revealed increased expression of chemokine and adhesion molecules, including *MCP1*, *ICAM1*, and *VCAM1*, in APOE knockout cells (Fig. S2E). Taken together, our results thus indicate that, APOE-deficient cardiomyocytes are more vulnerable and fragile than wild-type cells, in both non-inflammatory and inflammatory settings.

To further elucidate molecular mechanisms underlying the detrimental effects of APOE deficiency we had observed in human cardiovascular cells, we performed genome-wide transcriptomic sequencing for the three cardiovascular cell types. APOE deletion resulted in marked changes in gene expression in all three cell types, and the changes were mainly cell-type specific (Figs. 2A, S3A–C and Table S1). For example, of the 2,359 genes that are specifically dysregulated in *APOE*^−/−^ hVECs, the majority of upregulated genes are associated with NF-kappa B signaling pathway while downregulated genes are related to DNA repair. In contrast, out of 3,179 *APOE*^−/−^ hVSMC-specific dysregulated genes, upregulated genes are related to oxidative stress and downregulated genes are associated with vascular smooth muscle cell development. In hCMs, the number of differentially expressed genes (DEGs) was much lower, indicating that post-mitotic hCMs in culture are much less vulnerable to APOE depletion. The hCM-specific upregulated genes in *APOE*^−/−^ hCMs are mainly associated with negative regulation of chromosome organization, and the downregulated genes are mainly involved in regulation of cellular lipid catabolic process (Fig. 2B). Interestingly, a proportion of DEGs (23 upregulated and 15 downregulated genes) were present in all three cell types deficient in APOE. Gene Ontology (GO) term analysis of these DEGs revealed that upregulated genes are mainly related to inflammatory response, for example *APP*, which encodes the amyloid precursor protein, a transmembrane precursor protein that undergoes proteolysis to generate amyloid-beta peptides, known to play roles in CVDs, Alzheimer’s disease and aging. Downregulated common genes were enriched in tube morphogenesis and cell migration, as exemplified by *SEMA3C*, which encodes the neurovascular guiding molecule semaphorin 3C, that promotes migration and invasion, and may facilitate angiogenesis (Hao and Yu, 2018), and *SPRY1*, which encodes a cysteine-rich protein that plays an important role in cell proliferation, differentiation, migration and apoptosis (Lv et al., 2022) (Fig. 2B). Notably, the top commonly upregulated gene was the long noncoding RNA (lncRNA) maternally expressed gene 3 (*MEG3*) (Fig. 2C). Previous studies suggested that *MEG3* is involved in senescence and apoptosis of vascular cells and myocardial ischemia-reperfusion injury, and that inhibition of *MEG3* alleviates cardiac fibrosis in mice (Li et al., 2021). However, the transcriptional regulation and functional roles of *MEG3* in human cardiovascular cells remain unclear.

**Figure 2.**
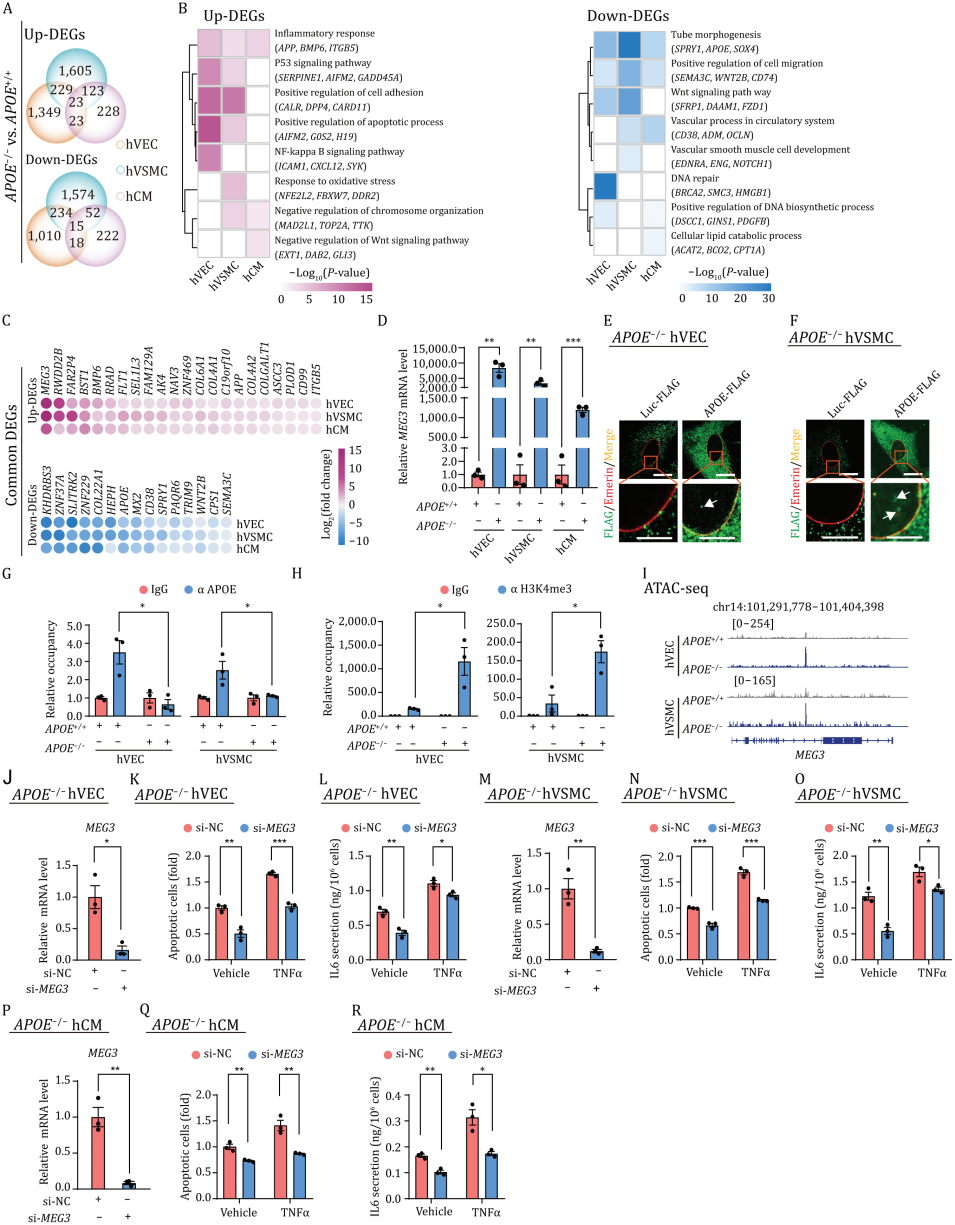
**APOE deficiency-induced aberrant expression of MEG3 has adverse effects on cardiovascular cells.** (A) Venn diagram showing the number of common and specific differentially expressed genes (DEGs) in APOE-deficient cardiovascular cells compared to their controls (*APOE*^−/−^ vs. *APOE*^+/+^). Up-DEGs, upregulated DEGs. Down-DEGs, downregulated DEGs. (B) Heatmaps showing Gene Ontology (GO) term and pathway enrichment analysis for up-DEGs (left) and down-DEGs (right) of indicated cell types (*APOE*^−/−^ vs. *APOE*^+/+^). The color keys from white to purple or blue indicate low to high enrichment levels. (C) Point plot showing common DEGs across three cell types (*APOE*^−/−^ vs. *APOE*^+/+^). The color key from blue to purple indicates log_2_(fold change) from low to high. (D) qRT-PCR analysis of the expression levels of *MEG3* in hVECs, hVSMCs and hCMs. Statistical results are presented as the means ± SEM. *n* = 3 independent experiments. **P < 0.01, ***P < 0.001. (E) Immunofluorescence analysis of FLAG and Emerin in *APOE*^−/−^ hVECs transduced with lentiviruses expressing Luc-FLAG or APOE-FLAG. The white arrow indicates nucleus-localized APOE. (F) Immunofluorescence analysis of FLAG and Emerin in *APOE*^−/−^ hVSMCs transduced with lentiviruses expressing Luc-FLAG or APOE-FLAG. The white arrows indicate nucleus-localized APOE. (G) ChIP-qPCR-based enrichment analysis of APOE within the promoter regions of *MEG3*. Statistical results are presented as the means ± SEM. *n* = 3 independent experiments. *P < 0.05. (H) ChIP-qPCR-based enrichment analysis of H3K4me3 within the promoter regions of *MEG3*. Statistical results are presented as the means ± SEM. *n* = 3 independent experiments. *P < 0.05. (I) Representative tracks showing the increased ATAC-seq signals within the gene body region of *MEG3* in APOE-deficient hVECs and hVSMCs compared to their controls (*APOE*^−/−^ vs. *APOE*^+/+^). (J) qRT-PCR analysis of the expression levels of *MEG3* in *APOE*^−/−^ hVECs transfected with si-NC or si-*MEG3*. Statistical results are presented as the means ± SEM. *n* = 3 independent experiments. *P < 0.05. si-NC, negative control siRNA. si-*MEG3*, siRNA for *MEG3*. (K) Flow cytometry-based apoptosis detection of hVECs transfected with si-NC or si-*MEG3* in the presence or absence of TNFα treatment. Statistical results are presented as the means ± SEM. *n* = 3 biological repeats. **P < 0.01, ***P < 0.001. si-NC, negative control siRNA. si-*MEG3*, siRNA for *MEG3*. (L) ELISA analysis for secreted IL6 in culture medium of hVECs transfected with si-NC or si-*MEG3* in the presence or absence of TNFα treatment. Statistical results are presented as the means ± SEM. *n* = 3 biological repeats. *P < 0.05, **P < 0.01. (M) qRT-PCR analysis of the expression levels of *MEG3* in *APOE*^−/−^ hVSMCs transfected with si-NC or si-*MEG3*. Statistical results are presented as the means ± SEM. *n* = 3 independent experiments. **P < 0.01. (N) Flow cytometry-based apoptosis detection of hVSMCs transfected with si-NC or si-*MEG3* in the presence or absence of TNFα treatment. Statistical results are presented as the means ± SEM. *n* = 3 biological repeats. ***P < 0.001. (O) ELISA analysis for secreted IL6 in culture medium of hVSMCs transfected with si-NC or si-*MEG3* in the presence or absence of TNFα treatment. Statistical results are presented as the means ± SEM. *n* = 3 biological repeats. *P < 0.05, **P < 0.01. (P) qRT-PCR analysis of the expression levels of *MEG3* in *APOE*^−/−^ hCMs transfected with si-NC or si-*MEG3*. Statistical results are presented as the means ± SEM. *n* = 3 independent experiments. **P < 0.01. (Q) Flow cytometry-based apoptosis detection of hCMs transfected with si-NC or si-*MEG3* in the presence or absence of TNFα treatment. Statistical results are presented as the means ± SEM. *n* = 3 biological repeats. **P < 0.01. (R) ELISA analysis for secreted IL6 in culture medium of hCMs transfected with with si-NC or si-*MEG3* in the presence or absence of TNFα treatment. Statistical results are presented as the means ± SEM. *n* = 3 biological repeats. *P < 0.05, **P < 0.01.

By qRT-PCR, we confirmed that *MEG3* was strongly upregulated in all the three cell types lacking APOE (Fig. 2D). Given that APOE was previously reported to be localized both in the cytoplasm and nucleus, and involved in epigenetic regulation and transcriptional regulation (Zhao et al., 2022), we suspected that APOE may be directly involved in regulation of *MEG3* expression. To explore this possibility, we first validated that APOE was detectable both in the cytoplasm and nucleus in hVECs and hVSMCs (Figs. 2E, 2F, S3D and S3E). ChIP-qPCR experiments further showed that APOE was associated with the promoter of *MEG3* in *APOE*^+/+^ cells, but this interaction was abrogated in hVECs or hVSMCs with APOE-depleted (Fig. 2G). Consistent with a transcriptional activation of *MEG3*, APOE deficiency resulted in increased enrichment of H3K4me3 in the promoter region of *MEG3* (Fig. 2H). Moreover, assay for transposase-accessible chromatin with high-throughput sequencing (ATAC-seq) revealed an increase in ATAC signals at the gene body region of *MEG3* in APOE-deficient cells compared to controls (Figs. 2I and S3F). Conversely, reintroduction of APOE into APOE-deficient cells suppressed the aberrant *MEG3* expression (Fig. S4A and S4B). Taken together, these data pointed to potential mechanism in which the APOE protein functions as a transcriptional repressor to regulate the expression of *MEG3*.

Next, to investigate whether *MEG3* functions as a downstream mediator of the detrimental effects caused by APOE deficiency, we knocked down *MEG3* in APOE-deficient hVECs, hVSMCs and hCMs, respectively (Fig. 2J, 2M and 2P). We observed that knockdown of *MEG3* was able to partially rescue the apoptosis and the expression of pro-inflammatory factors caused by APOE deficiency (Figs. 2K, 2L, 2N, 2O, 2Q, 2R and S4C–I). Likewise, when we re-introduced APOE or silenced *MEG3* in the presence of TNFα, we found that both approaches also attenuated the APOE deficiency-induced phenotypes (Figs. 2K, 2L, 2N, 2O, 2Q, 2R and S4C–I).

In summary, based on investigations using *in vitro* assays on human stem cell-derived models, we propose a crucial role for APOE in safeguarding diverse human cardiovascular cells from apoptosis and chronic inflammation. Mechanistically, our data support that APOE functions as a human cardiovascular cell protector, at least partly through transcriptionally repressing *MEG3*, whose activation is associated with CVD-like phenotypes. Our characterization of a novel protective mechanism for APOE in human cardiovascular cells provides fresh insights into CVD mechanisms and may help inform development of intervention strategies for cardiovascular disorders. Further studies are needed to distinguish which form of APOE, extracellular secreted form, cytoplasmic APOE or nuclear APOE is more specifically involved in the regulation of *MEG3* and the protection of cardiovascular cells.

## Supplementary Material

pwad017_suppl_Supplementary_Materials

pwad017_suppl_Supplementary_Table_S1

pwad017_suppl_Supplementary_Table_S2

## Footnotes

This work was supported by the National Key Research and Development Program of China (nos. 2020YFA0804000, 2022YFA1103700, 2020YFA0112200, 2021YFF1201005, 2022YFA1103800, the STI2030-Major Projects-2021ZD0202400), the National Natural Science Foundation of China (nos. 81921006, 82125011, 92149301, 92168201, 91949209, 92049304, 92049116, 32121001, 82192863, 82122024, 82071588, 32000500, 82271600, 32100937), the Strategic Priority Research Program of the Chinese Academy of Sciences (no. XDA16000000), the Program of the Beijing Natural Science Foundation (no. Z190019), CAS Project for Young Scientists in Basic Research (nos. YSBR-076, YSBR-012), The Pilot Project for Public Welfare Development and Reform of Beijing-affiliated Medical Research Institutes (no. 11000022T000000461062), Youth Innovation Promotion Association of CAS (nos. E1CAZW0401, 2022083), Young Elite Scientists Sponsorship Program by CAST (nos. YESS20200012, YESS20210002), the Informatization Plan of Chinese Academy of Sciences (nos. CAS-WX2021SF-0301, CAS-WX2022SDC-XK14, CAS-WX2021SF-0101), and the Tencent Foundation (no. 2021-1045).

We are grateful to Lei Bai, Ruijun Bai, Qun Chu, Xiangmei Jin, Luyang Tian, Jing Lu and Ying Yang for their administrative assistance. We thank Qing Meng (IOZ, CAS) for their assistance in flow cytometry analyses. We also thank the Biorender platform (biorender.com) which was used for schematic diagram.

The authors declare no competing interests.

All institutional and national guidelines for the care were followed.

All data associated with this study are present in the paper or the Supplementary Materials. The RNA-seq and ATAC-seq data obtained in the study have been deposited in the Genome Sequence Archive in the National Genomics Data Center, Beijing Institute of Genomics (China National Center for Bioinformation) of the Chinese Academy of Sciences, under accession number HRA003816.

G.H.L., J.Q., W.Z. and Q.L. conceived the work and supervised the experiments. H.Z. and Y.Z. performed the phenotypic and mechanistic analyses. K.Y. and Q.J. performed bioinformatic analyses. H.L., Z.W. and H.Z. performed the RNA-seq and ATAC-seq library construction. G.H.L., J.Q., W.Z., S.M., H.Z., K.Y., Z.W. and S.W. performed the data analysis. G.H.L., J.Q., W.Z., M.S., H.Z., Q.J., Z.W., S.W., and K.Y. performed manuscript writing, review, and editing. All authors reviewed the manuscript.

Conflict of interest: None declared.
