# Supplementary material for: APOE-mediated suppression of the lncRNA MEG3 protects human cardiovascular cells from chronic inflammation
Source: Protein Cell. 2023 Apr 3;14(12):908–13. doi: 10.1093/procel/pwad017 (PMC10691847; doi:10.1093/procel/pwad017)
Supplement: pwad017_suppl_Supplementary_Materials [file pwad017_suppl_supplementary_materials.pdf]

Supplemental Materials

Materials and methods

Cell culture

The hESCs (Line H1, WiCell Research Institute) were cultured on mitomycin C-inactivated MEF feeder layers in culture medium containing DMEM/F-12 (Thermo Fisher Scientific), 20% knockout serum replacement (Thermo Fisher Scientific), 1% nonessential amino acids (NEAAs) (Thermo Fisher Scientific), 1% GlutaMAX (Thermo Fisher Scientific), 1% penicillin-streptomycin (Thermo Fisher Scientific), 10 ng/ml basic fibroblast growth factor (bFGF) (Joint Protein Central), and 55 μM β-mercaptoethanol (Gibco), or on Matrigel-coated plates with mTeSR medium(Zhao et al., 2022). The hVSMCs were cultured in the presence of 50% Neurobasal (Gibco), 50% DMEM/F12 medium (Thermo Fisher Scientific), 1× N2 (Gibco), 1× B27 (Gibco), 10 ng/mL PDGF-AB (Peprotech) 55 μM β-mercaptoethanol (Gibco) and 1% penicillin-streptomycin (Thermo Fisher Scientific) in culture medium. The hVECs were cultured in an EGM-2 (Lonza) medium supplemented with 50 ng/mL VEGFA165 (HumanZyme), 20 ng/mL FGF2 (Joint Protein Central) and 10 nM SB431542 (Selleck). The hCMs were cultured in RPMI1640 medium (Gibco) containing S12 (plus insulin) medium. The S12 medium was an albumin-free and chemically defined supplement for cardiac differentiation including antioxidants, chemicals, protein, and fatty acid (Zhao et al., 2017). No mycoplasma contamination was observed during cell culture.

Generation of hVECs from hESCs

hVECs were differentiated from hESCs as previously described (Yan et al., 2019). Briefly, the hESC clones were picked onto 6-well plates coated with matrigel and cultured in mTeSR medium. On the second day, the medium was replaced with EGM-2 medium (Lonza) contained 3 μM CHIR99021 (Selleck), 4 ng/mL FGF2 (Joint Protein Central), 25 ng/mL BMP4 (R&D) and 3 μM IWP2 (Selleck) for 3 days. Then, the cells were cultured in EGM-2 medium supplemented with 50 ng/mL VEGFA165 (HumanZyme), 20 ng/mL FGF2 (Joint Protein Central) and 10 ng/mL IL6 (Peprotech) for a further 3 days. hVECs were incubated with anti-human CD34-FITC (BD biosciences, 555821, 1:200), anti-human CD201-PE (BD Biosciences, 557950, 1:200), and anti-human CD144-APC (BD Biosciences 561567, 1:200) and subsequently detected by flow cytometry (BD FACSAria IIIu).

Generation of hVSMCs from hESCs

hVSMC differentiation was performed as described previously (Ling et al., 2019; Wang et al., 2022). In brief, hESCs were dissociated into single cells using TrypLE Express (Gibco) and then plated into 6-well plates coated with matrigel at a density of 3×10^5 per well. The cells were cultured for 1 day in mTeSR with 10 μM Y-27632 (Selleck), and then cultured for 3 days in culture medium supplemented with 8 μM CHIR99021 (Selleck) and 25 ng/mL BMP4 (R&D). Ultimately, the cells were cultured in N2B27 medium supplemented with 10 ng/mL PDGF-AB (Peprotech) and 2 ng/mL Activin A (HumanZyme) for an additional 2 days and then incubated with anti-human CD140b-PE (BD Biosciences, 558821, 1:200) via flow cytometry (BD FACSAria IIIu).

Generation of hCMs from hESCs

Differentiation of hESCs into hCMs was performed as previously described (Zhang et al., 2022). The hESCs were separated into small colonies with 0.5 mM EDTA (Sigma, EDS-500G) and cultured on matrigel coated 24-well plates in mTESR medium. When the density of hESCs reached 80% to 90% confluence, the medium was changed to a differentiation medium composed of RPMI1640 and S12 (without insulin). 3 μM CHIR99021 was supplemented to the medium on day 0. After 24 hours, the medium was replaced to differentiation medium. From days 3-5, the medium was supplemented with 5 μM IWR-1. The medium was changed to RPMI1640 supplemented with S12 (with insulin) from day 5 to day 11. To purify hCM, the medium was changed to RPMI1640 (without glucose) supplemented with S12 (with insulin) and lactate every two days beginning on day 12.

TNFα treatment

Recombinant Human TNFα (Peprotech) was diluted to a concentration of 1 μg/mL with PBS. The hVSMCs and hVECs were seeded in 6-well plates at a density of 2×10^5 per well until reaching 70-80% confluence, and then treated with 20 ng/mL TNFα for 24 hours. The hCMs were treated in the similar way except for being cultured in 24-well plates with a density of 1×10^5 per well.

Flow cytometry-based detection of apoptosis

Apoptosis assays were performed using the Annexin V-FITC/PI Apoptosis Detection Kit (Vazyme Biotech Co., Ltd) based on the manufacturer's instructions. Cells were cultured in 12-well plates, after treatment with TNFα for 24 hours, the cells were harvested and washed twice in ice-cold PBS. Then, approximately 3×10^5 cells were resuspended in 100 μL 1 × binding buffer containing 5 μL propidium iodide (PI) and 10 μL Annexin V-FITC. The sample was vortexed and incubated at room temperature for 15 min in the dark. After adding another 400 μL 1 × binding buffer to each sample apoptotic rate was analyzed by flow cytometry (BD FACS Calibur), and the data were analyzed using FlowJo V10.

Flow cytometry-based detection of hVEC surface markers

The adherent cells were digested into single cells and centrifuged at 1,000 × rpm for 5 min at room temperature. After washing with PBS solution, the single cell suspension was adjusted to a concentration of 1×10^6 cells/ml. The cells were incubated with anti-human CD144-PE (BD Biosciences, 561714, 1:200) and anti-human CD31-FITC (BD Biosciences, 557508, 1:100) at room temperature in the dark for 30 min. Cells were then washed with PBS and re-suspended by 10% FBS in PBS. Samples were detected by flow cytometry, and the data were analyzed using FlowJo V10.

IL6 enzyme-linked immunosorbent assay (ELISA)

To determine the level of IL6 secreted by hVECs, hVSMCs or hCMs, 1×10^5 cells were seeded onto six-well plates and cultured with culture medium. After incubation for 2 days, the cell supernatant was collected and centrifuged to remove cells and then stored at -80 ℃. Then, the protein level of secreted IL6 in supernatant was analyzed using specific IL6 enzyme-linked immunosorbent assay kits (Biolegend) according to the manufacturer's instructions.

Western blot assay

Cells were washed with PBS and lysed in 1 x SDS lysis buffer (62.5 mM Tris-HCl, pH 6.8, 2% (wt/vol) SDS) for 10 min at 105 ℃ in a metal bath. Protein concentration was determined by the bicinchoninic acid (BCA) assay (BCA02; Dingguo Biotechnology). Equal quantities of protein (20 μg/lane) were isolated by 12% sodium dodecyl sulfate (SDS) polyacrylamide gels and transferred to polyvinylidene fluoride (PVDF) membranes (Merck Millipore). The membranes were blocked with 5% non-fat milk (BBI Life Sciences Corporation) at room temperature for 1 h. Then, the membranes were incubated with primary antibodies, including APOE (Santa Cruz Biotechnology, sc-13521, 1:500 dilution), at 4 ℃ overnight, β-Tubulin antibody (Immunoway, YM3030, 1:3,000 dilution) was used as a loading control. After washing three times with Tris Buffered Saline with Tween 20 (TBST), the membrane was incubated for 1 hour at room temperature with a secondary antibody coupled to horseradish peroxidase (HRP) (Jackson ImmunoResearch, 115-035-003, 1:5,000 dilution). Semi-quantification of blots was performed by ImageJ 1.47v analysis software for comparison.

Immunofluorescence assay

For immunofluorescence staining assay, cells seeded on microscope coverslips were washed with PBS three times, fixed with 4% formaldehyde for 15 min and permeated with 0.1% Triton X-100 for 10 min. Subsequently, the cells were washed with PBS three times and blocked with 10% donkey serum (Jackson ImmunoResearch) for 1 h at room temperature, Afterwards, the cell were incubated with primary antibodies at 4 ℃ overnight. Following incubation with fluorescence-labeled secondary antibodies at room temperature for 1 h, Then the cells were mounted in the mounting medium (Vector Laboratories) and the pictures were taken with a Zeiss LSM 900 confocal system. For the immunofluorescent staining, the antibody used as follows: anti-SM22 (Abcam, ab14106; 1:200 dilution), anti-Calponin (Dako, M3556; 1:200 dilution), anti-vWF (Dako, A0082; 1:200 dilution), anti-human CD31-FITC (BD Biosciences, 557508, 1:100 dilution), anti-Ki67 (Vector Laboratories, ZA0731; 1:500 dilution), anti-cTnT (Abcam, ab8295; 1:200 dilution).

Tube formation assay

hVECs (2×10^4 cells/well) were seeded on a 24-well plate coated with 200 μL matrigel (BD Biosciences) and cultured with 100 μL regular culture medium After incubation at 37°C for 8 h, and then stained by Calcein-AM (Y-D0041, Med Chem Express). Five randomly selected fields of view were captured using a fluorescent microscope (Olympus). The number of total tube joints was automatically analyzed using ImageJ 1.47v software (NIH).

Colony formation assay

Cells were treated with indicated conditions and then plated in 12-well plates (2,000/well). After cultured for 14 days, the cells were washed twice with PBS, fixed in 4% paraformaldehyde (PFA) for 30 min and incubated with crystalline violet solution (Biohao) for 30 min at room temperature. After washing with water, the images were acquired by Epson Perfection V370 Photo. Cell colonies were quantified in five different fields, and the mean value was calculated by ImageJ 1.47v software (NIH).

Transwell assay

Briefly, 2×10^4 cells from each group were resuspended in serum-free culture medium and plated in the upper Transwell chamber that had been pre-coated with 0.1% gelatin (Sigma, V900863-500G) at 37 ℃ overnight. A total of 500 μL normal culture medium was added to the lower chamber and cultured for 24 hours in 5% CO_2 humidified incubator. After incubation, the Transwell chamber were fixed with 4% paraformaldehyde and stained with crystalline violet solution for 30 min at room temperature. After being washed three times with PBS, picture was obtained by light microscopy and the number of migrating cells was calculated using ImageJ 1.47v software (NIH).

Small-interfering RNA (siRNA)-mediated knockdown of *MEG3*

For siRNA-mediated gene knockdown, siRNAs targeting *MEG3* (si-*MEG3*) and non-specific control siRNAs (si-NC) were synthesized by RiboBio (Guangzhou, China). The cells were transfected with si-*MEG3* and si-NC respectively using Lipofectamine RNAi-MAX Transfection Reagent (Thermo Fisher Scientific) according to the manufacturer's instructions. The transfected cells were collected 48 hours post-transfection for subsequent assays.

RNA extraction and real-time PCR

Total cellular RNA was extracted using the TRIzol Reagent (Invitrogen, CA) according to manufacturer's information. Quantitative RT-PCR was used to detect gene expression using the standard protocols on the ABI 7500 Real-Time PCR Detection system. GAPDH was the internal control. Primers were described in Supplementary Table 2. Data were analyzed using GraphPad Prism 8 software.

ChIP-qPCR analysis

For the ChIP-qPCR analysis, 2×10^6 hVECs and hVSMCs were hyperlinked in 1% formaldehyde (Sigma-Aldrich) at room temperature for 10 min, and then quenched with 0.125 M glycine (VWR International) at room temperature for 5 min. Next, cells were lysed on ice for 10 min and chromatin was clipped into fragments between 100-500 bp in target peak size with a Covaris S220 focused sonicator (Covaris). The supernatant was collected to incubate with APOE antibody (Santa Cruz, sc-13521) or H3K4me3 antibody (Abcam, ab8580) conjugated to DynabeadsTM Protein A (Thermo Fisher Scientific, 10006D) overnight at 4 ℃. Normal mouse IgG (Santa Cruz, sc-69786) or normal rabbit IgG (Cell Signaling Technology, 2729S) was used as the negative control. Subsequently, reversed cross-linking was carried out at 68 ℃ for 2 h by thermostat, and DNA isolation was completed by phenol-chloroform-isopentyl ester extraction and ethanol precipitation. The acquired DNA fragments were then analyzed by qPCR. All primers for this paper are described in Supplementary Table 2.

RNA-seq data processing

For RNA-seq raw data, sequences with adaptors and low-quality reads were removed by Trim Galore (version 0.6.6). Clean data was mapped to the UCSC human hg19 reference genome by STAR (version 2.7.1a) software (Dobin et al., 2013) with default parameters. The reads mapped to each gene were calculated using featureCounts (version 2.0.1) (Liao et al., 2014). To visualize the RNA-seq signals, RPKM (Reads Per Kilobase per Million mapped reads) were calculated for each 10 base pair (bp) using bamCoverage function in deepTools (version 3.5.1) software. Differentially expressed genes (DEGs) were calculated using the R package DESeq2 (version 1.30.1) (Love et al., 2014) with the cutoff of adjusted *P*-value less than 0.05 and $|\log_2$(fold

change)| more than 0.5. GO (Gene Ontology) terms and pathways enrichment analysis was performed by Metascape (Zhou et al., 2019). The DEGs are listed in Supplementary Table 1.

ATAC-seq data processing

For ATAC-seq data analysis, low-quality reads and adapters were first removed by Trim Galore (version 0.6.6), and clean reads were mapped to the UCSC human hg19 genome using Bowtie2 (version 2.4.5) (Langmead and Salzberg, 2012) with default parameters. Duplicated reads were removed by MarkDuplicates.jar program in Picard (version 2.27.1) and sorted by SAMtools (version 1.6) (Li et al., 2009). To minimize the effect of sequencing bias and depth, two replicates for each sample were merged, and high-quality reads were randomly sampled for further analysis. To determine the ATAC-seq signals, RPKM were calculated for each 10 bp using bamCoverage function in deepTools (version 3.5.1) software (Ram ŕez et al., 2016).

Statistical Analysis

Data are presented as the mean ± SEM. GraphPad Prism 8 software was used for two-tailed Student's *t*-test analysis. P value < 0.05, P value < 0.01 and P value < 0.001 were considered statistically significant (*, **, ***).

Data Availability

The RNA-seq and ATAC-seq data obtained in the study have been deposited in the Genome Sequence Archive in the National Genomics Data Center, Beijing Institute of Genomics (China National Center for Bioinformation) of the Chinese Academy of Sciences (Chen et al., 2021), under accession number HRA003816.

Supplemental Figure Legends

Figure S1. APOE is dispensable for cardiovascular cell differentiation of hESCs.

(A) Flow cytometry-based detection of canonical hVEC surface markers (CD31, CD144) in $APOE^{+/+}$ hVECs and $APOE^{-/-}$ hVECs. Statistical results are presented as the means ± SEM. *n* = 3 biological repeats ns, not significant.

(B) Immunofluorescence analysis of canonical hVEC markers CD31 and vWF, Nuclei were incubated with Hoechst 33342. Scale bar, 25 μm. Statistical results are presented as the means ± SEM. *n* = 3 biological repeats. ns, not significant.

(C) Flow cytometry-based detection of canonical hVSMC surface markers (CD140b) in $APOE^{+/+}$ hVSMCs and $APOE^{-/-}$ hVSMCs. Statistical results are presented as the means ± SEM. *n* = 3 biological repeats ns, not significant.

(D) Immunofluorescence analysis of canonical hVSMC markers Calponin and SM22, Nuclei were incubated with Hoechst 33342. Scale bar, 50 μm. Statistical results are presented as the means ± SEM. *n* = 3 biological repeats. ns, not significant.

(E) Immunofluorescence analysis of canonical hCM markers cTnT. Scale bar, 25 μm. Statistical results are presented as the means ± SEM. *n* = 3 biological repeats. ns, not significant.

Figure S2. APOE deficiency leads to reduced stress tolerance in cardiovascular

cells.

(A) Clonal expansion assay for *APOE*$^{+/+}$ hVECs and *APOE*$^{-/-}$ hVECs in the presence or absence of TNFα treatment. Statistical results are presented as the means ± SEM. n = 3 biological repeats. ns, not significant, ***, $P < 0.001$.

(B) qRT-PCR analysis of the expression levels of *VCAM1, ICAM1, MCP1* in hVECs in the presence or absence of TNFα treatment. Statistical results are presented as the means ± SEM. n = 3 independent experiments. *, $P < 0.05$, **, $P < 0.01$.

(C) Clonal expansion assay for *APOE*$^{+/+}$ hVSMCs and *APOE*$^{-/-}$ hVSMCs in the presence or absence of TNFα treatment. Statistical results are presented as the means ± SEM. n = 3 biological repeats. **, $P < 0.01$.

(D) qRT-PCR analysis of the expression levels of *VCAM1, ICAM1, MCP1* in hVSMCs in the presence or absence of TNFα treatment. Statistical results are presented as the means ± SEM. n = 3 independent experiments. *, $P < 0.05$, **, $P < 0.01$.

(E) qRT-PCR analysis of the expression levels of *VCAM1*, *ICAM1*, *MCP1* in hCMs in the presence or absence of TNFα treatment. Statistical results are presented as the means ± SEM. n = 3 independent experiments. *, $P < 0.05$, **, $P < 0.01$.

Figure S3. RNA-seq and ATAC-seq analysis for *APOE*$^{+/+}$ and *APOE*$^{-/-}$ cardiovascular cells.

(A) Euclidean distance analysis for RNA-seq data reproducibility. The color keys of the Euclidean distance from blue to white indicate strong to weak correlations.

(B) Principal component analysis (PCA) for RNA-seq data for indicated cell types.

(C) Representative tracks of RNA-seq showing expression levels of *APOE* in indicated cell types.

(D) Western blot analysis of APOE in *APOE*$^{-/-}$ hVECs transduced with lentiviruses expressing Luc-FLAG or APOE-FLAG.

(E) Western blot analysis of APOE in *APOE*$^{-/-}$ hVSMCs transduced with lentiviruses expressing Luc-FLAG or APOE-FLAG.

(F) Euclidean distance analysis of ATAC-seq data reproducibility in hVECs and hVSMCs. The color key of the Euclidean distance from blue to white indicate strong to weak correlations.

Figure S4. *MEG3* functions as a potential mediator for the damage effects of APOE deficiency.

(A) qRT-PCR analysis of the expression levels of *MEG3* in *APOE*$^{-/-}$ hVECs transduced with lentiviruses expressing Luc-FLAG or APOE-FLAG. Statistical results are presented as the means ± SEM. n = 3 independent experiments. *, $P < 0.05$.

(B) qRT-PCR analysis of the expression levels of *MEG3* in *APOE*$^{-/-}$ hVSMCs transduced with lentiviruses expressing Luc-FLAG or APOE-FLAG. Statistical

results are presented as the means ± SEM. *n* = 3 independent experiments. *, P < 0.05.

(C) qRT-PCR analysis of the expression levels of *IL6*, *VCAM1* in *APOE*$^{-/-}$ hVECs transduced with lentiviruses expressing Luc-FLAG or APOE-FLAG. Statistical results are presented as the means ± SEM. *n* = 3 independent experiments. *, P < 0.05.

(D) Flow cytometry-based apoptosis detection of *APOE*$^{-/-}$ hVECs transduced with lentiviruses expressing Luc-FLAG or APOE-FLAG. Statistical results are presented as the means ± SEM. *n* = 3 biological repeats. **, P < 0.01.

(E) qRT-PCR analysis of the expression levels of *IL6*, *VCAM1* in *APOE*$^{-/-}$ hVSMCs transduced with lentiviruses expressing Luc-FLAG or APOE-FLAG. Statistical results are presented as the means ± SEM. *n* = 3 independent experiments. *, P < 0.05, **, P < 0.01.

(F) Flow cytometry-based apoptosis detection of *APOE*$^{-/-}$ hVSMCs transduced with lentiviruses expressing Luc-FLAG or APOE-FLAG. Statistical results are presented as the means ± SEM. *n* = 3 biological repeats. *, P < 0.05.

(G) qRT-PCR analysis of the expression levels of *IL6*, *VCAM1* in *APOE*$^{-/-}$ hVECs transfected with si-NC or si-*MEG3* in the presence or absence of TNFα treatment. Statistical results are presented as the means ± SEM. *n* = 3 independent experiments. *, P < 0.05, **, P < 0.01.

(H) qRT-PCR analysis of the expression levels of *IL6*, *VCAM1* in *APOE*$^{-/-}$ hVSMCs transfected with si-NC or si-*MEG3* in the presence or absence of TNFα treatment. Statistical results are presented as the means ± SEM. *n* = 3 independent experiments. *, P < 0.05, **, P < 0.01.

(I) qRT-PCR analysis of the expression levels of *IL6*, *VCAM1* in *APOE*$^{-/-}$ hCMs with si-NC or si-*MEG3* in the presence or absence of TNFα treatment. Statistical results are presented as the means ± SEM. *n* = 3 independent experiments. *, P < 0.05, **, P < 0.01.

Supplementary Table Legends

Supplementary Table 1. Differentially expressed genes (DEGs) in APOE-deficient cardiovascular cells compared to their controls (*APOE*$^{-/-}$ vs. *APOE*$^{+/+}$).

Supplementary Table 2. Primers used for qRT-PCR and ChIP-qPCR analysis.

References

Chen, T., Chen, X., Zhang, S., Zhu, J., Tang, B., Wang, A., Dong, L., Zhang, Z., Yu, C., Sun, Y., *et al.* (2021). The Genome Sequence Archive Family: Toward Explosive Data Growth and Diverse Data Types. Genomics Proteomics Bioinformatics 19, 578-583.

Dobin, A., Davis, C.A., Schlesinger, F., Drenkow, J., Zaleski, C., Jha, S., Batut, P., Chaisson, M., and Gingeras, T.R. (2013). STAR: ultrafast universal RNA-seq aligner. Bioinformatics 29, 15-21.

Langmead, B., and Salzberg, S.L. (2012). Fast gapped-read alignment with Bowtie 2. Nat Methods 9, 357-359.

Li, H., Handsaker, B., Wysoker, A., Fennell, T., Ruan, J., Homer, N., Marth, G., Abecasis, G., and Durbin, R. (2009). The Sequence Alignment/Map format and SAMtools. Bioinformatics 25, 2078-2079.

Liao, Y., Smyth, G.K., and Shi, W. (2014). featureCounts: an efficient general purpose program for assigning sequence reads to genomic features. Bioinformatics 30, 923-930.

Ling, C., Liu, Z., Song, M., Zhang, W., Wang, S., Liu, X., Ma, S., Sun, S., Fu, L., Chu, Q., *et al.* (2019). Modeling CADASIL vascular pathologies with patient-derived induced pluripotent stem cells. Protein Cell 10, 249-271.

Love, M.I., Huber, W., and Anders, S. (2014). Moderated estimation of fold change and dispersion for RNA-seq data with DESeq2. Genome Biol 15, 550.

Ramírez, F., Ryan, D.P., Grüning, B., Bhardwaj, V., Kilpert, F., Richter, A.S., Heyne, S., Dündar, F., and Manke, T. (2016). deepTools2: a next generation web server for deep-sequencing data analysis. Nucleic Acids Res 44, W160-165.

Wang, S., Cheng, F., Ji, Q., Song, M., Wu, Z., Zhang, Y., Ji, Z., Feng, H., Belmonte, J.C.I., Zhou, Q., *et al.* (2022). Hyperthermia differentially affects specific human stem cells and their differentiated derivatives. Protein Cell 13, 615-622.

Yan, P., Li, Q., Wang, L., Lu, P., Suzuki, K., Liu, Z., Lei, J., Li, W., He, X., Wang, S., *et al.* (2019). FOXO3-Engineered Human ESC-Derived Vascular Cells Promote Vascular Protection and Regeneration. Cell Stem Cell 24, 447-461.e448.

Zhang, Y., Zheng, Y., Wang, S., Fan, Y., Ye, Y., Jing, Y., Liu, Z., Yang, S., Xiong, M., Yang, K., *et al.* (2022). Single-nucleus transcriptomics reveals a gatekeeper role for FOXP1 in primate cardiac aging. Protein & Cell.

Zhao, H., Ji, Q., Wu, Z., Wang, S., Ren, J., Yan, K., Wang, Z., Hu, J., Chu, Q., Hu, H., *et al.* (2022). Destabilizing heterochromatin by APOE mediates senescence. Nature Aging 2, 303-316.

Zhao, J., Cao, H., Tian, L., Huo, W., Zhai, K., Wang, P., Ji, G., and Ma, Y. (2017). Efficient Differentiation of TBX18(+)/WT1(+) Epicardial-Like Cells from Human Pluripotent Stem Cells Using Small Molecular Compounds. Stem Cells Dev 26, 528-540.

Zhou, Y., Zhou, B., Pache, L., Chang, M., Khodabakhshi, A.H., Tanaseichuk, O., Benner, C., and Chanda, S.K. (2019). Metascape provides a biologist-oriented

resource for the analysis of systems-level datasets. Nat Commun 10, 1523.
